# Prospective, comparative evaluation of a deep neural network and dermoscopy in the diagnosis of onychomycosis

**DOI:** 10.1371/journal.pone.0234334

**Published:** 2020-06-11

**Authors:** Young Jae Kim, Seung Seog Han, Hee Joo Yang, Sung Eun Chang

**Affiliations:** 1 Department of Dermatology, Asan Medical Center, University of Ulsan College of Medicine, Seoul, Korea; 2 Department of Dermatology, I Dermatology Clinic, Seoul, Korea; University of North Carolina at Chapel Hill, UNITED STATES

## Abstract

**Background:**

Onychomycosis is the most common nail disorder and is associated with diagnostic challenges. Emerging non-invasive, real-time techniques such as dermoscopy and deep convolutional neural networks have been proposed for the diagnosis of this condition. However, comparative studies of the two tools in the diagnosis of onychomycosis have not previously been conducted.

**Objectives:**

This study evaluated the diagnostic abilities of a deep neural network (http://nail.modelderm.com) and dermoscopic examination in patients with onychomycosis.

**Methods:**

A prospective observational study was performed in patients presenting with dystrophic features in the toenails. Clinical photographs were taken by research assistants, and the ground truth was determined either by direct microscopy using the potassium hydroxide test or by fungal culture. Five board-certified dermatologists determined a diagnosis of onychomycosis using the clinical photographs. The diagnosis was also made using the algorithm and dermoscopic examination.

**Results:**

A total of 90 patients (mean age, 55.3; male, 43.3%) assessed between September 2018 and July 2019 were included in the analysis. The detection of onychomycosis using the algorithm (AUC, 0.751; 95% CI, 0.646–0.856) and that by dermoscopy (AUC, 0.755; 95% CI, 0.654–0.855) were seen to be comparable (Delong’s test; P = 0.952). The sensitivity and specificity of the algorithm at the operating point were 70.2% and 72.7%, respectively. The sensitivity and specificity of diagnosis by the five dermatologists were 73.0% and 49.7%, respectively. The Youden index of the algorithm (0.429) was also comparable to that of the dermatologists’ diagnosis (0.230±0.176; Wilcoxon rank-sum test; P = 0.667).

**Conclusions:**

As a standalone method, the algorithm analyzed photographs taken by non-physician and showed comparable accuracy for the diagnosis of onychomycosis to that made by experienced dermatologists and by dermoscopic examination. Large sample size and world-wide, multicentered studies should be investigated to prove the performance of the algorithm.

## Introduction

Convolutional neural network (CNN) is a type of deep-learning algorithm that resembles the organization of the visual cortex. CNN models have advanced dramatically in recent years, ultimately being able to demonstrate physician-level diagnostic accuracy in a variety of medical fields such as dermatology, particularly skin cancers [1–9]. However, most studies have had a retrospective design and whether these data can be reproduced in a real clinical setting has not been assessed in prospective studies. In clinical practice, photographs are not taken for the diagnosis of onychomycosis unless it is an atypical case. Therefore, most onychomycosis images in hospital archives show atypical scenarios, introducing inherent selection bias into retrospective studies due to missing data. The study reported here therefore collected data prospectively to reduce the risk of selection bias.

Dermoscopy has recently been considered as a useful non-invasive approach to the diagnosis of onychomycosis. Conventionally, direct microscopic examination with potassium hydroxide (KOH) and fungal cultures have been the main diagnostic tools. However, these techniques are complex, time-consuming, and may be distressing for the patient due to the need for scraping. Despite dermoscopy examination’s benefits, including being non-invasive and having a real-time application, well-trained personnel are required to make an accurate diagnosis. In this prospective study, we evaluated the diagnostic power of a deep neural network in comparison with diagnosis made by experienced dermatologists and dermoscopic examination.

## Materials and methods

A prospective, observational comparative study was conducted at a tertiary hospital between September 2018 and July 2019. The study design was approved by the Institutional Review Board of Asan Medical Center (IRB number: 2018–1368).

Patients presenting with a dystrophic toenail were enrolled. To perform KOH evaluation, fungal culture, dermoscopic examination, and algorithm analysis in the same nail, targeted toes were identified by a skin marker. Clinical photographs of the whole foot were taken by research assistants. Direct microscopy with KOH 40% and culture were performed to confirm the diagnosis in all cases. The ground truth was determined either by direct microscopy with KOH testing or by fungal culture.

Five board-certified dermatologists (with a mean of 5.6 years of experience) determined a diagnosis of onychomycosis using the clinical photographs. Dermoscopic examination was performed using established diagnostic criteria [10] by two board-certified dermatologists. All dermoscopic features were recorded on a 10-point scale.

**Fig 1:**
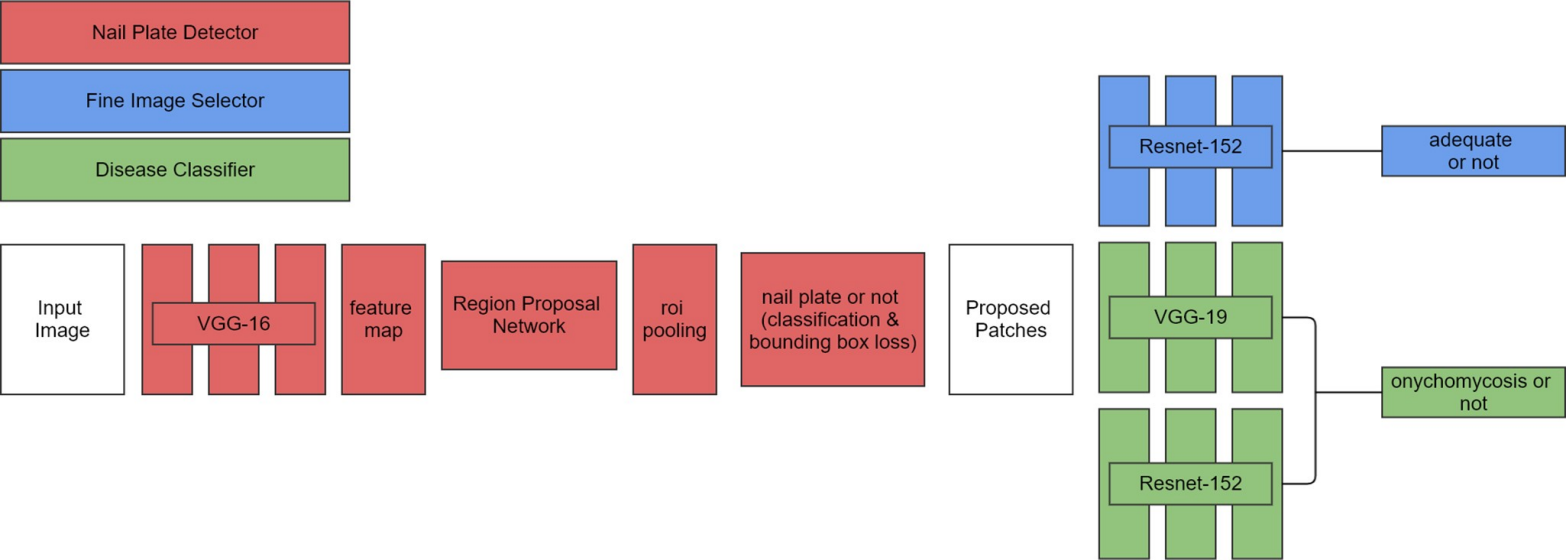
Architecture of the algorithm used in this study. Our algorithm comprised three parts: 1) the nail plate detector which detects nail plate from an unprocessed input image, 2) the fine image selector which excludes nail plate images with inadequate quality, and 3) the disease classifier which predicts the chance of onychomycosis. Using the Berkeley Vision and Learning Center (BVLC) deep learning framework Caffe, we fine-tuned the ImageNet pretrained models of ResNet-152 and VGG-19 for the onychomycosis classifier. We also fine-tuned the pretrained model of ResNet-152 for the fine image selector. For the nail plate detector, we used faster-RCNN (backbone network = VGG-16).

In a previous study [5], we created and released onychomycosis CNN models (see Data Availability and Fig 1); the same algorithm was used in this study without modification (http://nail.modelderm.com). The operating cut-off of the algorithm was obtained using the datasets (342 patients; 780 onychomycosis and 578 nail dystrophy images), which were used as the validation dataset in the previous study [5]. The optimal point that maximizes the sum of sensitivity and specificity was used as the operating cut-off threshold in this study.

Receiver operating characteristic (ROC) curves were drawn using each score of the algorithm and dermoscopic examination. The area under the curve (AUC; pROC package version, 1.15.3; R version 3.4.4) was calculated, and sensitivity, specificity, and Youden index score (sensitivity+specificity-100%) were compared between results of the algorithm, clinician evaluation, and dermoscopic examination. Wilcoxon rank-sum test was used to compare the variables. Delong’s test was performed to determine whether two ROC curves were statistically different. P-values <0.05 were considered statistically significant.

## Results

A total of 90 patients (mean age, 55.30±14.13 years; male, 44.3%) were included in the study (Table 1). KOH positivity was 84.2% (n = 48), culture positivity was 54.4% (n = 31), and positivity for both KOH and culture was 24.4% (n = 22). Since the ground truth was determined by either direct microscopy with KOH test or fungal culture, 63.3% of patients (n = 57) were diagnosed with onychomycosis.

**Table 1:** Demographics and clinical characteristics of patients.

Characteristics	Number of patients (%)	
	Onychomycosis	Onychodystrophy
	(n = 57)	(n = 33)
Age at diagnosis		
<19	0	0
19–39	8 (14.0)	5 (15.2)
40–59	23 (40.4)	14 (42.4)
≥ 60	26 (45.6)	14 (42.4)
Sex		
Male	30 (52.6)	9 (27.3)
Female	27 (47.4)	24 (72.7)
Location		
Left	22 (38.6)	14 (42.4)
Right	35 (61.4)	19 (57.6)
1^st^ toenail	53 (93.0)	26 (78.8)
2^nd^ toenail	1 (1.75)	2 (6.1)
3^rd^ toenail	1 (1.75)	0
4^rd^ toenail	0	1 (3.0)
5^th^ toenail	1 (1.75)	0
1^st^ finger nail	0	0
2^nd^ fingernail	1 (1.75)	0
3^rd^ fingernail	0	3 (9.1)
4^th^ fingernail	0	1 (3.0)
5^th^ fingernail	0	0
Types of onychomycosis		
DLSO	53 (93.0)	-
WSO	1 (1.7)	-
PSO	2 (3.5)	-
TDO	1 (1.7)	-
Nail involvement area		
Less than 1/4 of total nail	23 (40.4)	7 (21.2)
1/4 < area < 1/2 of total nail	14 (24.6)	14 (42.4)
1/2 < area < 3/4 of total nail	4 (7.0)	5 (1.5)
More than 3/4 of total nail	16 (28.1)	7 (21.)
KOH positivity	48 (84.2)	-
Culture positivity	31 (54.4)	-
Both positivity	22 (24.4)	-

Abbreviation: DLSO, distal and lateral subungual onychomycosis; WSO, white superficial onychomycosis; PSO, proximal subungual onychomycosis; TDO, total dystrophic onychomycosis

**Fig 2:**
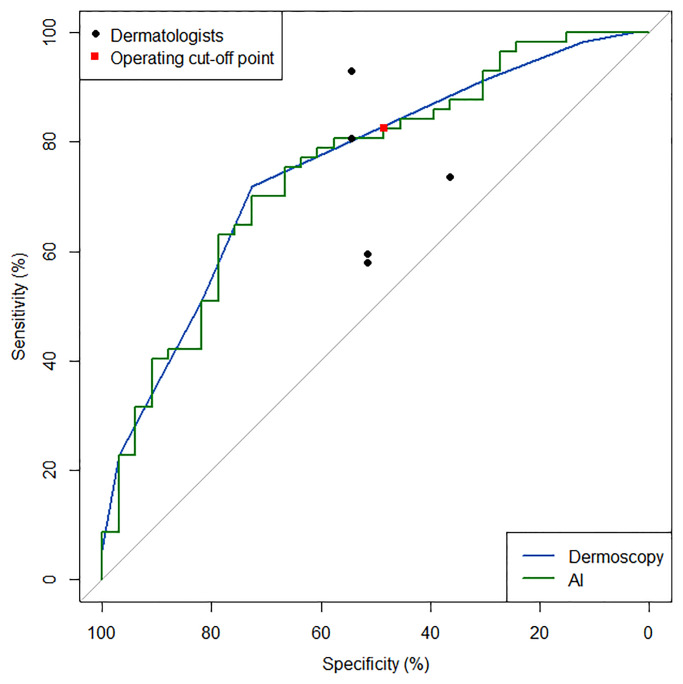
Receiver operating characteristic curves of the algorithm and the dermoscopic examination. The area under the curve (AUC) value of the algorithm was 0.751 (95% CI, 0.646–0.856), whereas the AUC value of dermoscopic examination was 0.755 (95% CI, 0.654–0.855). The results of the reader test are shown as circles (board-certified dermatologists).

The AUC value of the algorithm was 0.751(95% CI, 0.646–0.856), and the sensitivity/specificity of the algorithm at the cut-off threshold were 70.2/72.7% (Fig 2). The AUC value of dermoscopic examination was 0.755(95% CI, 0.654–0.855), and the sensitivity/specificity at the optimal operating point of the dermoscopic examination were 72.7/72.9%, respectively. Delong’s test showed no significant difference between the ROC curves of the algorithm and dermoscopic diagnosis (P = 0.952).

The mean sensitivity and specificity of diagnosis by five board-certified dermatologists were 73.0%±14.7% and 49.7%±7.6%, respectively. The mean Youden index of the five board-certified dermatologists was 0.230±0.176, which was comparable to that of CNN (0.429) using Wilcoxon rank-sum test (P = 0.667).

The positive predictive value/negative predictive value of the algorithm were 73.4%(95% CI, 61.5–82.7)/61.5%(95% CI, 35.5–82.3), and those of dermoscopic examination were 69.3%(95% CI, 58.2–78.6)/66.7%(95% CI, 41.7–84.8), and those of the five dermatologists were 76.8%±8.4% and 56.9%±15.5%, respectively.

Lastly, we examined whether antifungal medication was prescribed on the first day of visit. Among 90 patients, 84 (93.3%) were prescribed antifungal medication. All 57 patients of onychomycosis patients were prescribed antifungal medication, although only six (18. 2%) patients of onychodystrophy patients were not prescribed. This means 27 of 33 patients (81.8%) with onychodystrophy were wrongly prescribed antifungal medication at the first visit.

## Discussion

Onychomycosis, a fungal infection of the nail unit, is a widespread disease, with a reported annual prevalence of 2.18–13.8% in the USA [11]. This common nail disorder accounts for approximately 40% of all nail disorders [12] and fungal infection contributes to 0.15% of the global burden of disease measured in disability-adjusted life years [13]. Despite its high prevalence and clinical importance, it is challenging for clinicians to diagnose onychomycosis due to its similarity to other nail disorders.

Traditionally, mycological diagnosis was made using KOH examination or fungal cultures. The sensitivity and specificity of these tests were estimated to be 52.5–81.8% and 72.0–100%, respectively for KOH, and 57.0–59.0% and 82.0–100%, respectively, for fungal culture [12,14,15]. However, the two tests require the use of specific equipment and are time-consuming, particularly culture, which requires at least 4 weeks’ incubation. New diagnostic tools involving histopathologic examination using Periodic acid-Schiff staining of nail clippings have shown greater sensitivity (88.2–93.1%) but cannot provide an immediate diagnosis in the clinical setting [16].

The algorithm used in the current study demonstrated comparable accuracy to the diagnosis of dermoscopic features. Unlike KOH and dermoscopic examination, which are time-consuming and must be carried out by well-trained personnel, diagnosis using CNN can be made using photographs taken by non-physicians in a real-time setting.

**Fig 3:**
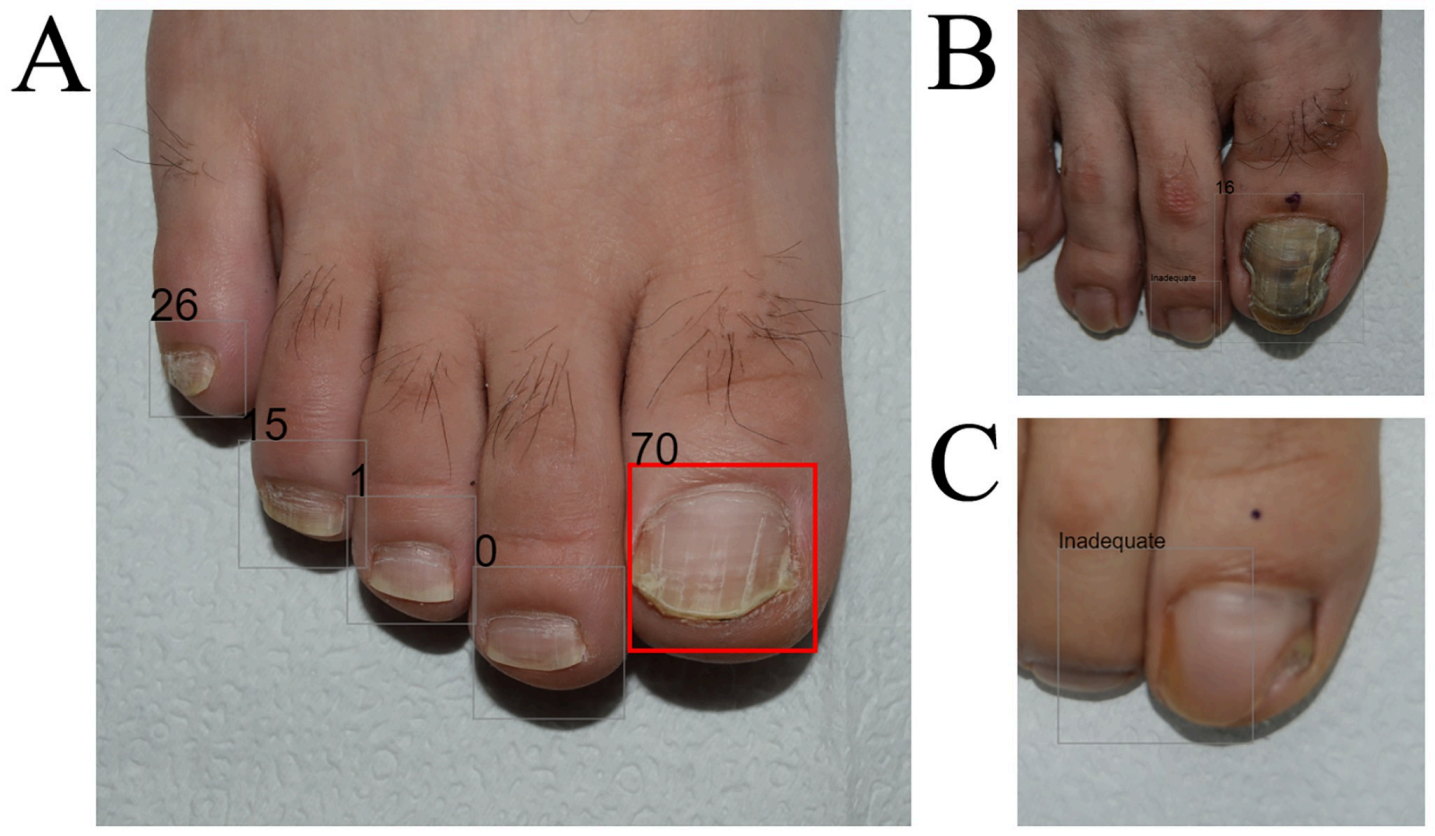
Examples of diagnostic output images. (A) Correct example; a 24-year-old male, confirmed as having onychomycosis by KOH examination and culture. AI made an accurate diagnosis of onychomycosis using this image, whereas two of the five dermatologists misdiagnosed the case as onychodystrophy in the reader test. The rectangle was colored when the onychomycosis output was higher than the operating cut-off threshold (29.3; range 0–100). (B) Incorrect example; a 26-year-old male, confirmed as having onychomycosis by the KOH examination. AI made an inaccurate diagnosis of onychodystrophy using this image, whereas all five dermatologists correctly diagnosed the condition as onychomycosis. (C) Inadequate quality image; a 49-year-old female, confirmed as having onychomycosis by both KOH examination and culture study. AI first recognized the nail plate, and then the onychomycosis classifier determined whether the nail plate image was onychomycosis or not. With the low-quality, unfocused nail image, AI could not recognize the features properly, resulting in an unreliable diagnostic prediction.

However, the algorithm used here has several limitations. First, because this study was performed in a tertiary hospital, results with the cases in primary center should be further investigated in multicenter large studies. Second, the results can be significantly affected by the quality of the input images [5]. This has been demonstrated in the previous study, where poor-quality photographs were associated with less accurate diagnostic capabilities [5]. As shown in Fig 3C, failed cropping occurs if the photographs obtained by non-physicians are inadequate. Although an ancillary algorithm that can exclude inadequate photographs can accommodate this problem, the impact of image quality on diagnostic accuracy should be further assessed. Lastly, diagnostic approaches in a real practice setting should be processed after checking the clinical features of soles, all toenails, and past medical history.

Despite the growing requests for practical application in healthcare system, recent studies have raised concerns about deep learning algorithm. The systematic review of artificial intelligence studies warned that most trials for machine learning studies have potential high risk of bias, and recommended prospective design [17]. To date, only 9 prospective machine learning studies have been reported in all medical fields.

Unlike previous studies, our study is designed particularly for assisting non-dermatologists rather than dermatologic experts, and the algorithm is fully opened and accessible through the website. This aspect of our algorithm enables patients to screen their onychomycosis on a daily life without the help of the specialists. In addition, the algorithm can assist non-dermatologic physician to decide the necessity of antifungal medication, thus we expect to decrease the erroneous prescription of antifungal medication for onychodystrophy. When we analyzed the area involvement of nail, 65.0% of patients revealed nail involvement in less than half of total nail area (Table 1). Relatively higher frequency of mild cases in this study implies more beneficial value of our algorithm in patients’ daily self-practical application.

## Conclusion

In conclusion, as a standalone method, the algorithm used in this study was able to analyze photographs taken by non-physicians, demonstrating comparable diagnostic accuracy to that of experienced dermatologists and dermoscopic examination. Large, multinational, multicenter studies are warranted to further evaluate the performance of the algorithm.

## Supporting information

S1 Dataset Minimal data set. (PDF)
