# Correction: Prospective, comparative evaluation of a deep neural network and dermoscopy in the diagnosis of onychomycosis

**DOI:** 10.1371/journal.pone.0244899

**Published:** 2020-12-29

**Authors:** Young Jae Kim, Seung Seog Han, Hee Joo Yang, Sung Eun Chang

There is information missing from the Funding statement. The complete, correct Funding statement is as follows: This study was supported by a grant from 2018 Paul Janssen scholarship award to SEC. The funder had no role in study design, data collection and analysis, decision to publish, or preparation of the manuscript.
