# Supplementary material for: Prospective, comparative evaluation of a deep neural network and dermoscopy in the diagnosis of onychomycosis
Source: PLoS One. 2020 Jun 11;15(6):e0234334. doi: 10.1371/journal.pone.0234334 (PMC7289382; doi:10.1371/journal.pone.0234334)
Supplement: S1 Dataset — (PDF) [file pone.0234334.s001.pdf]

(Minimal Data Set)

Patient number	Sex	Age	Dermoscopy (0-10)	AI (0-100)	Ground truth (Pos=1, Neg=0)	Dermatologist1	Dermatologist2	Dermatologist3	Dermatologist4	Dermatologist5	Type	Invasion degree (A<1/4, 1/4≤B<1/2, 1/2≤C<3/4, 3/4≤D)
1	M	67	5	35.25	0	1	1	1	1	1	DSO	B
2	F	57	1	1.3	0	0	0	0	0	0	DSO	A
3	M	24	4	62.65	1	1	1	1	1	1	DSO	B
4	F	55	3	58.07	0	0	1	1	1	1	DSO	B
5	F	68	5	67.86	1	1	1	1	1	0	DSO	B
6	F	37	3	96.96	0	1	1	1	0	0	DSO	C
7	F	48	5	86.79	1	1	1	1	1	1	DSO	A
8	M	76	3	96.6	1	1	0	1	1	1	DSO	B
9	F	64	3	19.7	0	1	0	1	1	1	DSO	B
10	F	72	5	20.71	0	0	0	0	0	0	DSO	B
11	F	24	2	64.09	1	1	1	1	0	1	DSO	B
12	F	41	5	98.75	1	1	0	1	0	0	DSO	D
13	F	34	3	98.86	1	1	1	1	1	1	DSO	D
14	F	53	5	20.25	1	0	1	1	1	1	WSO	D
15	M	55	4	86.72	0	1	0	1	0	0	DSO	A
16	F	64	3	74.34	0	1	1	1	0	0	DSO	B
17	F	65	5	98.66	1	1	0	1	1	1	DSO	B
18	F	55	3	93.27	0	1	0	0	0	1	PSO	B
19	F	64	3	39.87	1	1	0	1	0	0	DSO	B
20	F	29	5	83.5	1	1	0	0	0	1	DSO	C
21	M	27	3	23.61	0	0	0	0	0	0	TSO	D
22	M	58	5	25.18	1	0	0	0	0	0	DSO	A
23	M	59	5	95.59	1	1	1	1	1	0	DSO	B
24	M	60	4	63.95	1	1	1	1	0	0	DSO	A
25	F	65	5	67.45	0	1	1	1	1	1	PSO	C
26	F	55	2	6.26	0	0	0	0	1	1	WSO	D
27	F	54	5	99.97	1	1	1	1	1	0	DSO	D
28	F	71	3	87.32	0	1	0	1	0	0	DSO	D
29	M	77	3	54.16	1	0	0	0	0	0	TSO	C
30	F	64	6	96.29	1	1	0	0	1	0	DSO	A
31	F	76	3	85.55	0	1	0	0	0	1	DSO	A
32	M	25	6	99.74	1	1	1	0	1	1	DSO	C
33	F	49	7	97.81	1	1	0	0	0	0	DSO	B
34	M	60	2	6.22	1	0	1	1	0	0	DSO	A
35	M	68	4	14.41	1	0	1	1	1	1	DSO	A
36	F	40	2	0.5	1	0	0	1	1	1	DSO	A
37	F	48	2	52.66	0	0	1	0	0	0	DSO	B
38	F	71	2	25.84	0	0	1	0	0	0	DSO	B
39	M	51	5	71.56	1	0	1	1	1	1	DSO	B
40	F	77	3	69.86	1	0	1	1	1	1	DSO	A
41	F	40	5	72.68	1	1	1	1	0	1	PSO	A
42	M	76	5	99.62	1	1	1	1	1	1	DSO	B
43	M	55	4	6.54	1	0	1	1	1	1	DSO	B
44	F	65	3	92.98	1	1	1	1	1	1	DSO	A
45	F	36	5	19.64	0	1	1	0	0	0	DSO	D
46	M	67	5	19.52	1	1	1	1	1	1	DSO	A
47	M	72	4	89.13	1	1	1	0	0	0	DSO	A
48	F	62	2	0.02	0	0	1	1	1	1	DSO	A
49	M	64	4	82.75	1	1	1	1	1	1	DSO	D
50	F	47	3	0.03	0	0	1	1	1	1	WSO	D
51	M	45	3	24.95	0	0	1	0	1	1	DSO	B
52	F	55	5	99.61	1	1	1	1	1	1	DSO	A
53	F	72	6	84.32	1	1	1	0	0	0	DSO	B
54	M	50	7	97.09	1	1	1	0	0	0	DSO	D
55	M	71	3	73.17	1	1	1	0	0	0	DSO	A
56	F	49	6	98.05	1	1	1	1	0	0	DSO	D
57	M	70	0	55.34	0	0	1	0	0	0	DSO	B
58	F	28	1	0	0	0	1	0	0	0	DSO	B
59	M	25	4	90.42	1	1	1	1	0	1	DSO	D
60	M	60	3	33.48	1	0	1	0	0	0	DSO	A
61	F	60	4	98.8	1	1	1	0	0	1	DSO	A
62	M	59	5	35.28	0	1	1	0	1	0	DSO	A
63	F	45	6	99.15	1	1	1	1	1	1	DSO	A
64	M	61	3	0.17	0	0	1	1	0	0	DSO	A
65	F	52	5	68.09	1	0	1	1	1	0	DSO	D
66	F	57	3	99.04	0	1	1	1	1	1	DSO	B
67	M	65	3	66.02	1	1	1	1	0	1	DSO	A
68	M	38	5	54.94	1	1	1	1	0	0	PSO	C
69	M	50	6	53.6	1	0	1	1	1	1	DSO	D
70	M	26	6	80.95	1	1	1	1	1	1	DSO	D
71	M	64	3	95.42	1	1	1	0	0	0	DSO	C
72	M	57	3	5.9	0	0	1	1	0	1	DSO	B
73	F	32	4	10.5	0	0	1	1	1	1	DSO	B
74	F	53	1	6.65	1	1	1	1	1	0	DSO	D
75	F	52	6	42.83	0	0	1	1	1	1	DSO	A
76	M	61	6	70.01	1	1	1	1	0	0	DSO	A
77	M	68	6	90.05	1	1	1	1	1	1	DSO	D
78	M	65	3	78.54	1	1	1	1	1	1	DSO	B
79	F	61	2	0.28	0	0	1	1	1	1	DSO	A
80	M	48	4	94.92	1	1	0	1	1	1	DSO	C
81	M	44	4	50.53	1	1	1	1	1	1	DSO	D
82	F	66	4	21.91	1	1	1	1	1	1	DSO	D
83	F	50	2	65.6	0	1	1	1	1	1	DSO	B
84	F	75	2	15.41	1	1	0	1	1	1	DSO	A
85	F	81	4	1.59	0	0	0	1	1	0	TDO	D
86	F	59	3	47.44	0	1	1	1	0	0	DSO	D
87	F	49	7	98.03	1	1	1	1	0	0	DSO	D
88	M	66	1	33.64	0	0	1	1	1	1	DSO	A
89	M	45	4	46.04	1	1	1	0	0	0	DSO	C
90	F	52	6	88.59	1	1	1	1	1	1	DSO	A
